# A Study of Methylene Blue Adsorption by a Synergistic Adsorbent Algae (*Nostoc sphaericum*)/Activated Clay

**DOI:** 10.3390/polym17152134

**Published:** 2025-08-04

**Authors:** Yakov Felipe Carhuarupay-Molleda, Noemí Melisa Ccasa Barboza, Sofía Pastor-Mina, Carlos Eduardo Dueñas Valcarcel, Ybar G. Palomino-Malpartida, Rolando Licapa Redolfo, Antonieta Mojo-Quisani, Miriam Calla-Florez, Rolando F. Aguilar-Salazar, Yovana Flores-Ccorisapra, Arturo Rojas Benites, Edward Arostegui León, David Choque-Quispe, Frida E. Fuentes Bernedo

**Affiliations:** 1 Basic Sciences Department, José María Arguedas National University, Andahuaylas 03701, Peru; raguilar@unajma.edu.pe (R.F.A.-S.); arojas@unajma.edu.pe (A.R.B.); ffuentes@unajma.edu.pe (F.E.F.B.); 2 Environmental Engineering, José María Arguedas National University, Andahuaylas 03701, Peru; sofiapastormina@gmail.com (S.P.-M.); ceduenas@unajma.edu.pe (C.E.D.V.); earostegui@unajma.edu.pe (E.A.L.); 3 Chemical Engineering Department, San Cristobal de Huamanga National University, Ayacucho 05000, Peru; ybar.palomino@unsch.edu.pe (Y.G.P.-M.); dinner.licapa@unsch.edu.pe (R.L.R.); 4 Agroindustrial Engineering, Universidad Nacional de San Antonio Abad del Cusco, Cusco 08000, Peru; antonieta.mojo@unsaac.edu.pe (A.M.-Q.); miriam.calla@unsaac.edu.pe (M.C.-F.); 5 Systems Engineering Department, Universidad Nacional José María Arguedas, Andahuaylas 03701, Peru; yflores@unajma.edu.pe; 6 Agroindustrial Engineering, José María Arguedas National University, Andahuaylas 03701, Peru; dchoque@unajma.edu.pe

**Keywords:** dye residues, optimal adsorption, nanoclay, adsorbent dose, spontaneous and stable process

## Abstract

Dye residues from the textile industry constitute a critical wastewater problem. This study aimed to evaluate the removal capacity of methylene blue (MB) in aqueous media, using an adsorbent formulated from activated and sonicated nanoclay (NC) and microatomized *Nostoc sphaericum* (ANS). NC was obtained by acid treatment, followed by activation with 1 M NaCl and sonication, while ANS was obtained by microatomization in an aqueous medium. NC/ANS was mixed in a 4:1 weight ratio. The NC/ANS synergistic adsorbent was characterized by the point of zero charge (PZC), zeta potential (ζ), particle size, FTIR spectroscopy, and scanning electron microscopy (SEM). NC/ANS exhibited good colloidal stability, as determined by pH_PZC_, particle size in the nanometer range, and heterogeneous morphology with functional groups (hydroxyl, carboxyl, and amide), removing between 72.59 and 97.98% from an initial concentration of 10 ppm of MB, for doses of 20 to 30 mg/L of NC/ANS and pH of 5 to 8. Optimal adsorption conditions are achieved at pH 6.8 and 32.9 mg/L of adsorbent NC/ANS. It was observed that the pseudo-first-order (PFO) and pseudo-second-order (PSO) kinetic models best described the adsorption kinetics, indicating a predominance of the physisorption process, with adsorption capacity around 20 mg/g. Isotherm models and thermodynamic parameters of adsorption, ΔS, ΔH, and ΔG, revealed that the adsorption process is spontaneous, favorable, thermodynamically stable, and occurs at the monolayer level, with a regeneration capacity of 90.35 to 37.54% at the fifth cycle. The application of physical activation methods, such as sonication of the clay and microatomization of the algae, allows proposing a novel and alternative synergistic material from organic and inorganic sources that is environmentally friendly and promotes sustainability, with a high capacity to remove cationic dyes in wastewater.

## 1. Introduction

The demand for synthetic dyes in various industries (textile, pharmaceutical, paper, food, among others) is increasing significantly worldwide, contaminating water resources through wastewater discharge [1]. Current regulations, such as the Environmental Quality Standards and the Maximum Permissible Levels in the case of Peru, are transversal; however, the lack of industrial water treatment plants aggravates the problem.

The annual production of industrial dyes amounts to thousands of tons. The most commonly used dyes are alcian blue, basic fuchsin, crystal violet, toluidine blue, and methylene blue (MB), which are cationic dyes. Other commonly used dyes are aniline blue, Congo red, methyl orange, acid black, and acid red, which are anionic dyes [2,3]. Although the use of anionic dyes is between 60% and 70% compared with cationic dyes, the latter cause greater environmental damage due to their low degradability, especially in water bodies. Therefore, several studies have focused on proposing materials that can remove these dyes, such as methylene blue [4,5,6,7,8,9,10,11,12].

MB is the most widely used dye and one of the most studied due to health side effects such as increased heart rate, vomiting, respiratory toxicity, mucous membrane irritation, diarrhea, and skin cancer [13]. Similarly, in aquatic environments, even at low concentrations, they inhibit the metabolic and photosynthetic processes of living organisms, causing deterioration in aquatic ecosystems [14,15].

Traditional methods for wastewater treatment, such as chemical precipitation, filtration, and reverse osmosis, are expensive, so the aim is to apply emerging methods that are easy to operate and economical, such as adsorption, which also allows the use of ecological adsorbents such as biomass waste and activated clay from sustainable, renewable, and abundant sources [14,16,17].

Several studies have been conducted on inorganic materials (mainly clays, zeolites, and metal oxides such as TiO_2_ and Fe_2_O_3_), organic materials (such as lignocellulosic biomass, activated carbon, chitosan, and their modifications), and synthetic materials (mainly polyacrylamide, alginates, polyethyleneimine, and ion exchange resins) for MB adsorption [6,18]. Organic materials are inexpensive and sustainable, but their capacity depends on the treatment, and their stability is limited. Inorganic materials are cheap and abundant, although sensitive to pH and generate non-treatable waste. Synthetic materials offer high efficiency and specificity but are expensive and can release toxic waste [19,20]. Furthermore, they are subject to limitations such as rapid saturation, dye desorption, and complex regeneration. Therefore, ensuring adequate adsorption depends on cost, efficiency, effluent conditions, and environmental sustainability criteria, and these aspects can be improved through the synergy of these individual materials [21,22,23,24].

Clay is used as an efficient alternative in the removal of anionic and cationic dyes due to its high ion exchange capacity and specific surface area, low cost, abundance, and eco-friendliness, although it has some disadvantages, such as complex regeneration and limited selectivity, as well as requiring chemical modification to optimize removal efficiency [25,26,27]. On the other hand, many materials of biological origin have been used for the removal of dyes in aqueous media, including algae such as Chlorella, Spirulina, and Sargassum, due to their functional groups (carboxyl, amino, and hydroxyl), offering advantages of biodegradability, environmental friendliness, recyclability, and promotion of green chemistry and contributing to sustainable development, although their advantages are limited by their limited stability, complex separation, and sensitivity to the pH and temperature of the aqueous medium [28,29]. Nostoc sphaericum is a high Andean algae that requires minimal nutrient demand, grows above 4000 m altitude, and has qualities that make its use as an adsorbent of cationic dyes potential due to its good stability in aqueous media, its anionic functional groups, and, above all, the extraction of its hydrocolloid does not require processes that involve the use of chemical substances [30,31,32].

The fusion of new adsorbent materials such as atomized *Nostoc sphaericum* (NS) and activated nanoclays (NC) is a promising proposal to improve the adsorption capacity of MB, due to their properties. NS is a new material in the world of adsorption; however, NC would stand out as a good adsorbent due to its surface area, structural properties, and functional groups of anionic character (such as -OH, -COOH, and -NH_2_), which can establish electrostatic attraction and bind cationic molecules [25,33].

Many studies of inorganic–organic materials in the adsorption of dyes confirm high percentages of contaminant removal in water, since they offer superior performance to their components due to their unique properties and structural diversity [34], making these materials potential low-cost and environmentally friendly adsorbents.

Obtaining the adsorbent from organic matter requires treatment and activation processes that optimize its removal capacity. Clay, an abundant and easily accessible material, has been shown in numerous studies to be suitable for the removal of dyes from wastewater. Similarly, *Nostoc sphaericum*, a seaweed available in high Andean lagoons during the rainy season, is a promising material for the removal of cationic dyes in aqueous media because it is composed primarily of proteins and polysaccharides, which contain negatively charged groups such as carboxyl, carbonyl, and hydroxyl groups [30,35,36,37]. Its advantage lies in its biodegradable and sustainable nature; however, it has limitations due to its limited reusability. The use of bio-based materials allows for the removal of MB above 90% due to their chemical composition, as demonstrated by chitosan, alginate, hydrogels, and modified cellulose [38,39,40].

Therefore, the study aimed to elaborate an NC/ANS adsorbent in different formulations and to evaluate the adsorption capacity of MB at different pH and adsorbent doses; likewise, to know its adsorption capacity, the adsorption kinetics were studied for optimum conditions.

## 2. Materials and Methods

### 2.1. Raw Materials and Reagents

Clay was collected from a quarry in the Huancabamba zone, Andahuaylas, Peru (13°43′58″ S, 73°20′38″ W, 3682 m of altitude). NS was collected at Huamanilla Lagoon, Andahuaylas, Peru, at coordinates 13°47′52″ S, 73°17′55″ W and 4251 m of altitude, during the rainy season of 2023.

Solutions of phosphoric acid (H_3_PO_4_) (Oxford, Maharashtra, India), methylene blue from Spectrum Chemical Mfg. Corp. (New Brunswick, NJ, USA), sodium chloride (NaCl), potassium bromide (KBr), and hydrochloric acid (HCl) (EMSURE, Darmstadt, Germany) were used.

### 2.2. Clay Treatment and Activation

**Figure 1:**
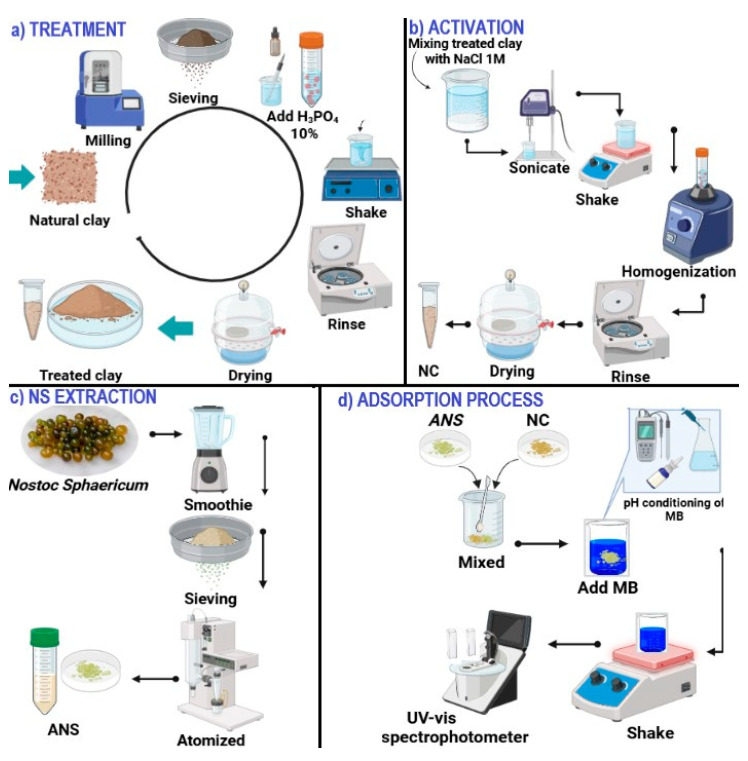
Methodologies and processes: (**a**) clay treatment, (**b**) clay activation, (**c**) *Nostoc sphaericum* extraction, and (**d**) MB adsorption process.

The ground and sieved clay was treated with 10% H_3_PO_4_, stirring at 300 rpm for 6 h. It was washed with distilled water until neutral and dried at 60 °C in a Binder Model BD56 oven (Tuttlingen, Germany), thus obtaining treated clay (Figure 1a). NaCl was then added at 1 M (5:1 *v*/*w*) and stirred at 200 rpm for 24 h, after which it was washed with ultrapure water until obtaining a conductivity <10 μS/cm. Then it was sonicated for 10 min at 740 W power, 750 kJ energy, and 40% amplitude in a Sonics VCX750 equipment (Newtown, CT, USA) and dried at 60 °C for 24 h. It was then ground in a planetary ball mill (PM10-Resch, Haan, Germany) at 3000 rpm for 5 min and sieved through a 45 µm mesh (model AS200, Resch, Haan, Germany), obtaining activated nanoclay (NC) [33] (Figure 1b).

### 2.3. Nostoc Sphaericum Extraction

The NS with an average moisture content of 98% was washed with abundant distilled water, then homogenized with distilled water (1:1 *w*/*w*) in a domestic blender, and sieved at 120 µm. It was then atomized in a Buchi mini spray dryer, model B-290 (Flawil, Switzerland) at 100 °C, air flow 650 L/h, 85% suction, and feed rate 5 mL/min to obtain atomized *Nostoc sphaericum* (ANS) (Figure 1c) [41].

### 2.4. Point of Zero Charge (PZC), Zeta Potential (ζ), and Particle Size Determination

For this, a 50 mL solution was prepared at pH 2 to 12, 0.05 g of adsorbent was added (1 ANS/4 NC by weight), and stirred at 60 rpm for 24 h at 20 °C. The initial and final pH values were then plotted to determine the PZC. The ζ was determined by dynamic light scattering (DLS) by preparing a suspension of 4 mg adsorbent in 50 mL ultrapure water, stirring at 1000 rpm for 5 min, and sonicating for 10 min. An aliquot was added to the size determinator (model ZSU3100, Malvern Instruments, Worcestershire, UK) at 25 °C, 632.8 nm, scattering angle of 14.14°, and electric field strength of 5 V/cm.

### 2.5. FTIR Analysis

Tablets of the NC, ANS, ANS/NC (1:4 *w*/*w*), and NC/ANS-MB (after adsorption) were prepared at 0.1% KBr. Readings were performed on the transmission module of the Nicolet IS50 FTIR spectrometer, Thermo Fisher (Waltham, MA, USA), with a wavelength of 4000–400 cm^−1^, a resolution of 8 cm^−1^, and 32 scans.

### 2.6. SEM and EDX Analysis

Samples of NC, ANS, ANS/NC (1:4 *w*/*w*), and NC/ANS-MB were fixed with carbon tapes, and their morphology was determined using a Prism E scanning electron microscope, Thermo Fisher (Massachusetts, MA, USA) at 25 kV acceleration and 1000× magnification. The elemental composition was also measured by X-ray energy dispersive spectroscopy in the same equipment. 

### 2.7. Effect of pH and Adsorbent Dosage

Solutions of 10 mg/L MB were prepared at pH 5, 6, and 8. 20, 30, and 50 mg/L of adsorbent (1 ANS/4 NC) were added to each solution and stirred at 60 rpm for 60 min (Figure 1d). Readings were taken in a UV–Vis spectrophotometer at 664 nm (model GENESYS, Thermo Fisher, Waltham, MA, USA). The standard curve (R^2^ = 0.9994) was performed previously.

The percentage removal rate (%R) and adsorption capacity were calculated using Equations (1) and (2) [34].

(1) $$\%R=\frac{C_{i}-C_{f}}{C_{i}}*100$$

(2) $$q_{e}=\frac{{(C}_{i}-C_{f})}{m}*V$$

where *C_i_* and *C_f_* are the initial and final concentrations of MB, respectively (mg/L); m is the mass of the adsorbent (g); *V* is the volume of the solution (L); *q_e_* is the adsorption capacity (mg/g); *R* is the percentage of removal (%).

### 2.8. Optimization of the Adsorption Process

Empirical models were constructed according to Equation (3). Linear, interaction, and quadratic models were proposed and evaluated using the Excel Solver application. The restrictions were the maximum and minimum pH value and adsorbent dose, while the objective function was to maximize R.

(3) $$Y=\beta_{0}+\sum \beta_{0}X_{i}+\sum \beta_{ii}X_{i}^{2}+\sum \beta_{ij}X_{i}X_{j}$$

where *Y* is the predicted response expressed in percentage (%), X_i_ and X_j_ are the independent variables (pH and adsorbent dose), *β*_0_ is the constant, and *βi*, *β_ii,_* and *β_ij_* are the linear, quadratic, and interaction coefficients of the independent variables, respectively.

### 2.9. Adsorption Kinetics Study

A 100 mL solution containing 10 mg/L of MB was prepared at the pH values and the optimum dosage. Adsorption was evaluated at different times (0, 5, 20, 40, 60, 100, 150, 200, 300, and 400 min) and under constant agitation at 60 rpm and 20 °C. An aliquot was taken to the spectrophotometer at 664 nm, and the concentration of MB was reported.

The adsorption kinetics were fitted using the model:

(a). Pseudo-first order (PFO) (Equation (5)) kinetic models [42,43,44].
  (4) $$\frac{{dq}_{t}}{dt}=k_{1}(q_{e}-q_{t}),$$

The integration of the equation, for time t = 0 to t = t, the concentration of MB in the adsorbent is q = 0 to q = qt, and has the form

(5) $$q_{t}=q_{e}(1-e^{-k_{1}t}),$$

(b). Pseudo second order (PSO) [45] (Equation (7))
  (6) $$\frac{{dq}_{t}}{dt}=k_{2}{\left(q_{e}-q_{t}\right)}^{2},$$

Considering the boundary conditions of the pseudo-first-order equation, we obtain

(7) $$q_{t}=\frac{q_{e}^{2}k_{2}t}{1+q_{e}k_{2}t}\;,$$

(c). Elovich model [46,47] (Equation (10))
  (8) $$\frac{{dq}_{t}}{dt}=\alpha \cdot {exp}^{-\beta q_{t}},$$
  when *q_t_* →0, $\frac{{dq}_{t}}{dt}$ → *α*, which is the initial adsorption rate (mg/g·min), and *β* is the sorption constant. Integrating and applying the limits for *t* (0,*t*) and *q_t_* (0,*q_t_*), Elovich’s model is
  (9) $$q_{t}=\frac{1}{\beta }\ln\left(1+\alpha \beta t\right),$$

For an equilibrium system, *t* >> $\frac{1}{\alpha \beta }$, the equation can be written as

(10) $$q_{t}=\frac{1}{\beta }\ln\left(\alpha \cdot \beta \right)+\frac{1}{\beta }\ln\left(t\right),$$

(d). Intraparticle diffusion (ID) [48,49] (Equation (12))

The description of intraparticle sorbate diffusion in the porous particle is based on Fick’s diffusion laws. The diffusion process considering sorbents can be described by the mass balance equation [48,49] in the form

(11) $$\frac{\partial C_{t}}{\partial t}=D\left(\frac{\partial^{2}C_{t}}{\partial x^{2}}+\frac{2}{x}\frac{\partial C_{t}}{\partial x}\right),$$

The probability of the adsorbate being transported from a concentrated zone to the adsorbent through diffusion is considered, this being the step that limits the speed in many adsorption processes, generally for discontinuous stirring processes, where adsorption varies almost proportionally to t^1/2^ instead of the contact time t [50], according to the following equation:

(12) $$q_{t}=k_{i}t^{1/2}+C,$$

(e). Avrami model

It is applied when it is suspected that the adsorption process involves complex mechanisms such as internal diffusion or surface heterogeneity, as well as the presence of multiple mechanisms or heterogeneous growth of adsorbed phases. It is represented by Equation (13) [51].

(13) $$q_{t}=q_{e}(1-e^{\left(-k_{Av}t^{n}\right)})$$

(f). Boyd model

It is used to predict the rate-limiting step of adsorption, whether it is by internal (intraparticle) or external (pellicular) diffusion. Its nonlinear form is shown in Equation (14) [52].

(14) $$F=1-\frac{6}{\pi^{2}}\sum_{n=1}^{\infty }\frac{1}{n^{2}}exp\left({-n}^{2}B_{t}\right)$$

A particular solution for Bt (a mathematical function of F) is represented by Equation (15), for F > 0.85.

(15) $$B_{t}=-0.4977-ln(1-F)$$

where *q_e_* is the equilibrium adsorption capacity (mg/g); *q_t_* is the adsorption capacity at one time (mg/g); *k*_1_ is the first-order kinetic constant (1/min); *k*_2_ is the second-order kinetic constant (g/mg·min); *t* is the time (minutes); *β* is the number of sites available for adsorption; *α* is the initial adsorption rate (mg/g); *k_i_* is the ID rate, and *C* is the constant ID rate (if C = 0, adsorption is minimal and diffusion within the adsorbent is the main factor; if C ≠ 0, adsorption occurs both on the surface and inside the adsorbent, and high values of C favor surface adsorption); *k_Av_* is the Avrami rate constant; *n* is the Avrami constant (if *n* = 1, one-dimensional adsorption; *n* = 2, two-dimensional adsorption; *n* = 3, three-dimensional adsorption; and *n* = 4, adsorption with spherical growth); F is the fraction adsorbed at time t (F = q_t_/q_e_); and Bt is the Boyd function.

### 2.10. Adsorption Equilibrium Study

MB solutions with concentrations ranging from 5 to 150 mg/L (adsorbate) were prepared, and the adsorbent was added under optimal adsorption conditions, stirring at 50 rpm for 60 min at 20, 25, 30, 35, and 40 °C. An aliquot was taken and analyzed in a UV–Vis spectrophotometer at 664 nm. The adsorption capacity was calculated using Equation (1). The data obtained were fitted to adsorption isotherm models.

Langmuir model (L)

The model (Equation (16)) indicates that the adsorption capacity increases with the equilibrium concentration until it reaches a saturation value corresponding to the total surface area of the adsorbent monolayer, and no further adsorption will occur [53].

The affinity between the adsorbate and the adsorbent is calculated using the Langmuir separation factor *R_L_* (Equation (17)). If *R_L_* > 1, it is unfavorable; if 0 < *R_L_* < 1, it is favorable (adsorbate retention occurs); if *R_L_* = 0, it is irreversible (the adsorbate is not easily desorbed and is permanently retained) [54].

Multilayer model (M)

The model (Equation (18)) proposes that in an adsorbent with a homogeneous surface, there are two adsorption sites in the system: the original sites of the solid surface for adsorption in the first layer and the adsorbate site for adsorption in the second and subsequent layers (multilayer) [55].

Freundlich model (F)

The model (Equation (19)) proposes that adsorption occurs on a heterogeneous adsorption surface (ideal adsorption) composed of active groups with different adsorption potentials and different adsorption energies [56,57].

Redlich–Peterson model (R–P)

The model (Equation (20)) explains the heterogeneity of the sorbent through the energy distribution of the active sites. The three-parameter model is formulated by combining the Langmuir and Freundlich models [58].

Temkin model (T)

The model (Equation (21)) assumes that the heat of adsorption in the adsorbent layers decreases linearly due to adsorbate–adsorbent interactions and that adsorption occurs with uniform binding energies up to a maximum energy. Its parameters allow the variation in adsorption energy to be determined [59].

Dubinin–Radushkevich model (D–R)

The isotherm (Equation (22)) explains adsorption as a Gaussian energy distribution on a heterogeneous and porous surface, free energy (*E*) (Equation (23)), that is, it is the energy required to remove molecules from the adsorbent surface. If E < 8 kJ/mol, physisorption predominates, and if 8 < E < 168 kJ/mol, adsorption is chemical. Adsorption occurs when the micropore of the adsorbent is filled with the dye molecule [60].

(16) $$q_{e}=\frac{q_{m}k_{L}C_{e}}{1+k_{L}C_{e}},$$

(17) $$R_{L}=\frac{1}{\left(1+k_{L}C_{0}\right)},$$

(18) $$q_{e}=\frac{q_{m}k_{1}C_{e}}{\left(1-k_{2}C_{e}\right)[1+\left(k_{1}+k_{2}\right)C_{e}]},$$

(19) $$q_{e}=k_{f}C_{e}^{1/n},$$

(20) $$q_{e}=\frac{k_{R}C_{e}}{1+a_{R}C_{e}^{g}},$$

(21) $$q_{e}=\frac{RT}{b_{t}}\ln(A_{t}C_{e}),$$

(22) $$q_{e}=q_{m}e^{-k_{DR}\varepsilon^{2}},$$

(23) $$E=\frac{1}{\sqrt{2k_{DR}}},$$

where *q_e_* (mg/g) is the equilibrium adsorption capacity; *k_L_* (L/mg) is the Langmuir constant; *C_e_* (mg/L) is the equilibrium adsorbate concentration; *q_m_* (mg/g) is the adsorption capacity of the monolayer; *k*_1_ (L/mg) is the adsorption affinity constant for the first layer; *k*_2_ (L/mg) represents the adsorption affinity for subsequent multilayers; *K_f_* (mg^1−1/n^/L^n^) and *n* are the Freundlich constants, indicators of adsorption capacity and adsorption intensity, respectively; *k_R_* (L/g) indicates the maximum adsorption capacity; *a_R_* (L/mg)^g^ is the constant parameter of the R–P model; *g* is the parameter that varies between 0 and 1; *R* is the universal gas constant (8.314 J/mol·K), *T* is the temperature (K), *b_T_* (J/mol) is the change in adsorption energy, *A_T_* (L/mg) is the maximum binding constant, *k_RD_* (mmol^2^/J^2^) is the constant of Dubinin–Radushkevich isotherm, and Ɛ is the Polanyi potential (J/mol).

The kinetic and isotherm models were fitted using nonlinear regression. *R*^2^ (Equation (24)), Average Relative Error (*ARE*) (Equation (25)), and Chi-square (*χ*^2^) (Equation (26)) were calculated to evaluate the accuracy of the models and minimize the fractional error [61].

(24) $$R^{2}=1-\frac{\Sigma_{\mathrm{i}=1}^{N}{{{(q}_{exp}-q}_{adj})v}^{2}}{\Sigma_{\mathrm{i}=1}^{N}{{{(q}_{exp}-\bar{q}}_{exp})}^{2}}$$

(25) $$ARE=\frac{100}{1^{v}}\Sigma_{\mathrm{i}=1}^{N}{\left|\frac{q_{adj}-q_{exp}}{q_{exp}}\right|}_{i}$$

(26) $$X^{2}=\Sigma_{\mathrm{i}=1}^{N}{\left|\frac{{{(q_{exp}-q}_{adj})}^{2}}{q_{adj}}\right|}_{i}$$

where *q_adj_* is the reported adsorption capacity, *q_exp_* is the experimental adsorption capacity, $\bar{q}$_exp_ is the mean of *q_exp_*, and N is the total number of values or estimates.

### 2.11. Adsorbent Regeneration Cycle

The method proposed by Auta et al. [62] was adapted. After the test, the adsorbent was recovered under optimum conditions by filtration at 0.22 µm, rinsed with plenty of distilled water, and dried at 60 °C. The recovered adsorbent was added to 100 mL of a 0.1 M HCl desorption solution and stirred continuously at 60 rpm for 60 min. Then, it was recovered by filtration, rinsed with deionized water, and dried. The adsorption process then proceeded. This operation was repeated five times.

### 2.12. Statistical Data Processing

The data, represented in triplicate, were presented as mean values, standard deviations, and coefficients of variability in dynamic tables and graphs. A two-factor ANOVA with interaction described in the Pareto diagram was applied. The Taylor plot was also used to analyze the fit and correlation of the kinetic models employing the mean square error. All analyses were performed at 5% significance. Excel spreadsheets, OriginPro 2025, and STATISCA 12.0 were used.

## 3. Results and Discussion

### 3.1. Point of Zero Charge and Zeta Potential

**Figure 2:**
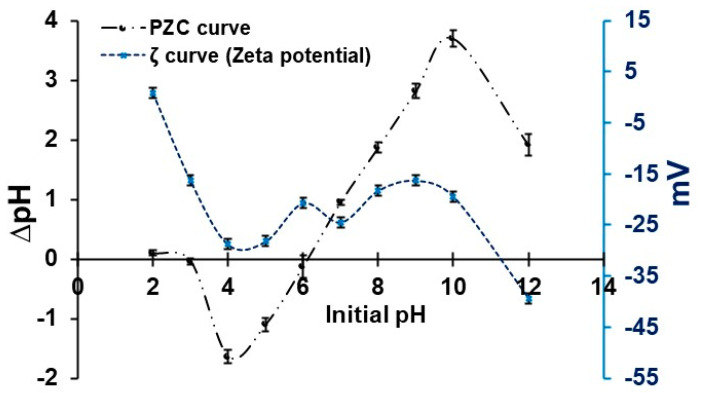
PZC and ζ for adsorbent NC/ANS.

The point of zero charge (PZC) indicates the pH at which the surface charge of the material is neutral. The adsorbent has two pH_PZC_ values around 3 and 6 (Figure 2). This fact would be due to the composition of the adsorbent, that is, it is composed of NC (inorganic material) and ANS (organic material). This phenomenon is because biomass materials, such as algae, generally have PZC values between 6 and 9, while inorganic materials, such as clays, have lower values, usually around 3. This difference is attributed to the functional groups in organic materials that can interact with protons, resulting in higher PZC values [33,63]. The adsorbent is negatively charged when the pH of the solution is higher than the pH_PZC_, which favors the adsorption of cationic dyes such as MB [64,65]. At a pH below 3, the surface of the adsorbent has a positive charge due to protonation (H^+^ ions); the increase in pH leads to a deprotonation of the surface [66,67,68]. Between pH 3 and 6, a transition occurs with a mixed charge distribution. In general, better adsorption of MB would occur at a pH higher than 6.

The ζ indicates the stability of the adsorbent in aqueous media. The values observed in Figure 2 report an average stability of the adsorbent between pH 3 and 10, with values ranging from −16.32 to −28.83 mV, with a significant decrease at pH 12 (−39.34 mV). Although at pH < 3 it tends to be positively charged, this would be due to the excess of hydrogen ions in the medium, which suggests low stability, and the NC/ANS would precipitate rapidly. Therefore, the copolymer would respond better under neutral and alkaline conditions. While at pH 2.1 (isoelectric point), the maximum aggregation or suspension of the adsorbent occurs, establishing the minimum electrostatic repulsion in the aqueous medium. The synergy of two compounds represents a greater stability to pH variations [69]. Variations in NC stability are related to particle size, chemical composition, hydration, charge density, and surface texture; in contrast, ANS stability is attributed to the presence of carboxyl, carbonyl, and hydroxyl groups [26,30,41,70]. The ζ is essential to determine the adsorption capacity, since it influences the interaction between the adsorbent particles and the dye molecules [71].

### 3.2. Adsorbent Particle Size

The particle size of the adsorbent varies between 532.17 and 5468 nm (Table 1). Two groups are observed at pH 4, 7, 9, and 12. This would be due to the aggregation of adsorbent particles and could be attributed to the composition of the NC and ANS due to the influence of their functional groups. At pH 2, the size is larger and decreases with increasing pH. In both algal biomass and clay, smaller particles provide a larger contact surface, which would improve the interaction with MB due to the greater availability of active sites [18,72]. Adsorption of a contaminant depends on the cation exchange capacity and surface charge of the adsorbent; however, a high surface charge can result in more dispersed particles and less aggregation of the contaminant, which can negatively affect adsorption efficiency [21,27].

### 3.3. Functional Group Analysis

**Figure 3:**
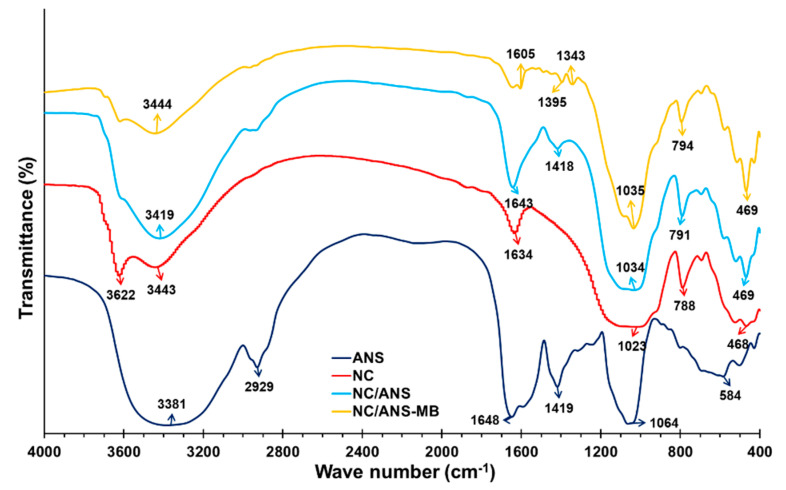
FTIR spectrum of NC, ANS, NC/ANS, and NC/ANS-MB.

The vibrations at the 3622 cm^−1^ peak in NC (Figure 3) correspond to the -OH stretching of the internal hydroxyl group in the form of Mg-OH-Al, Fe-OH-Al, and Al-OH-Al, present between the tetrahedral and octahedral sheets. While in ANS the peaks between 3300 and 3600 cm^−1^ indicate vibration of N-H deformations in amines and O-H stretching of hydroxyl groups [24,73,74,75], they also suggest the presence of hydrogen bonds due to the interaction between carbohydrates and water [35]. The peak around 2929 cm^−1^ in ANS indicates C-H stretching vibrations of methyl groups [76,77]. The peaks around 1600 cm^−1^ correspond to the amide group [78]. Around 1419 cm^−1^, C-H deformations, and C-N vibrations are found coming from the protein structures and nitrogenous compounds of ANS, while between 1300 and 1400 cm^−1^, it indicates symmetric stretching of COO^−^ groups [22,79].

On the other hand, the adsorbent (NC/ANS) presents new molecular interactions that are not found in its components at 1418 cm^−1^, which would correspond to negatively charged amino and carboxyl groups, while at 1034 cm^−1^ a peak with greater intensity is seen and would be due to the presence of negatively charged groups (C-O, C-O-C, C-OH of ANS, and Si-O of NC). This is corroborated by the high intensity of NC at 1023 cm^−1^ and ANS at 1064 cm^−1^, which suggests the interaction of the functional groups of NC and ANS, although no new functional groups would have been produced, because the preparation of the adsorbent was not through a chemical reaction or the intervention of another constituent. Likewise, the number of hydroxyl groups increases, showing greater intensity around 3400 cm^−1^. These identified groups intervene in the MB adsorption process, interacting with its positive groups (quaternary and tertiary amino) through electrostatic interactions, hydrogen bonds, and Van der Waals interactions, influencing the elimination of MB in aqueous medium [18,80,81,82]. This is evidenced in Figure 3, where peaks with less intensity are observed, which confirms the NC/ANS adsorbent capacity.

Likewise, peak displacement was observed around 3400 cm^−1^ on the adsorbent after adsorption (NC/ANS-MB) (Figure 3), indicating the adsorption of MB due to the formation of hydrogen bridges of the amino group of MB with the OH groups. Similarly, between 1340 and 1400 cm^−1^, displacement and decrease of the peak intensity were observed in NC/ANS-MB, due to interactions between the positively charged dimethylamino groups of MB and the negative sites of the adsorbent [18,83].

In the region between 450 and 580 cm^−1^, significant peak modifications are observed after adsorption, which is indicative of the significant adsorption of MB, mainly due to electrostatic interactions in the NC sheets and the amino groups of ANS with the thiazine conjugated system of MB [18,84].

### 3.4. EDX and SEM Analysis

**Figure 4:**
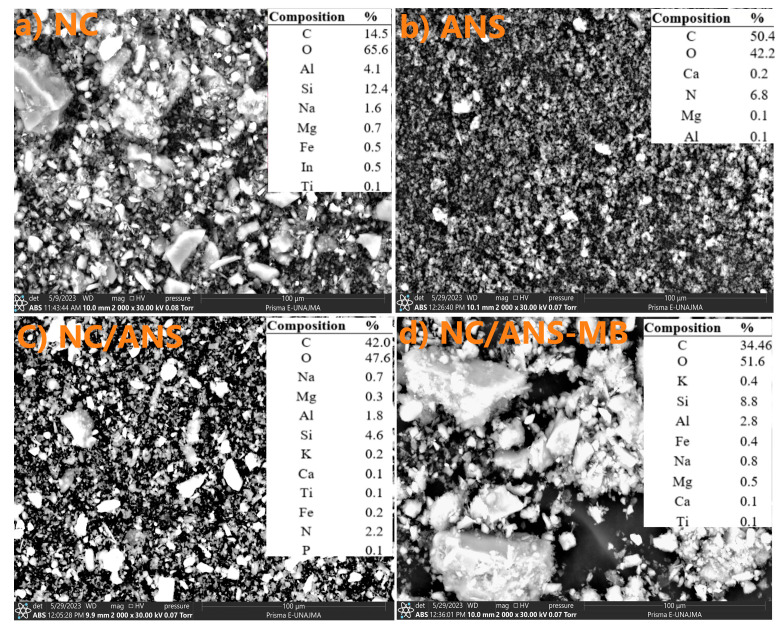
Morphology images of NC, ANS, NC/ANS, and NC/ANS-MB.

The NC morphology presents a high composition of O (65.6%), Si (12.4%), C (14.5%), and Al (4.1%), typical of aluminum silicates, quartz, and carbonates, as well as exchangeable cations such as Na, Mg, and Fe in lower percentages (Figure 4a). Likewise, ANS presented a homogeneous organic structure, rich in C (50.4%) and O (42.2%), with significant N content (6.8%) due to its high carbohydrate and protein content (Figure 4b). The mineral content of Ca, Mg, and Al in low proportions reflects its organic nature, that is, the microelements of this type of algae [85,86,87]. On the other hand, NC/ANS presented C (42.0%) and O (47.6%) (Figure 4c), which indicates that the adsorbent presents organic matter, composed of anionic groups (carbonyl, carboxyl, and amino) from the ANS and the carbonate and quartz from the NC, as evidenced by the FTIR analysis. The presence of Na is due to the activation of NC with NaCl.

Regarding morphology, it is observed that the clays present a heterogeneous surface, with fine particles of irregular shapes, including laminar and angular structures (Figure 4a). While ANS shapes are relatively spherical and more uniform, characteristic of materials with high hydrocolloid content [30]. Finally, NC/ANS-MB (Figure 4d) presented changes in surface morphology; these changes confirm the effectiveness of the dye’s adsorption. The combination of clay and Nostoc improves the adsorption capacity due to the increased surface area and functional groups available for interaction [88,89,90].

### 3.5. Effect of pH and NC/ANS Dose on MB Removal and Adsorption

**Figure 5:**
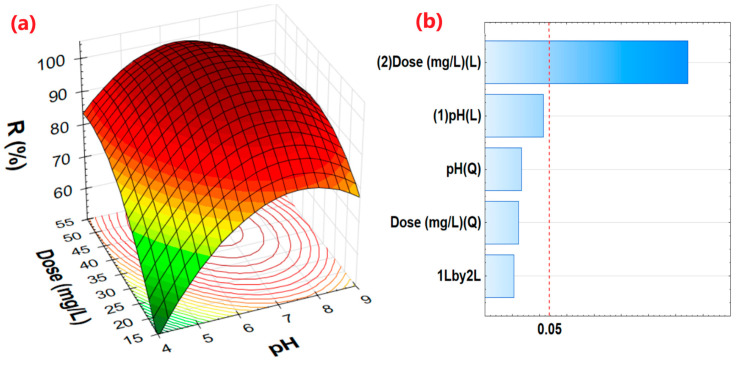
(**a**) Response surface for percent removal, (**b**) Pareto diagram.

The effect of pH and NC/ANS dose revealed that the highest %R of MB occurred in T6 (pH 6, 50 mg/L) with 97.98%. While T1 presented a higher adsorption capacity with 2.73 mg/g (pH 5, 20 mg/L), thus demonstrating the efficiency of the adsorbent. This fact indicates that, at pH levels ranging from slightly acidic to neutral, the adsorbent captures more MB molecules, as seen in Figure 5a, where the best removal is obtained in this limited pH area. Moreover, the removal percentage increases considerably with adsorbent dosage (Figure 5a), indicating that it has a significant effect on MB removal (Figure 5b). This is due to the presence of a larger number of active adsorption sites [19,91,92]. Similar behavior was reported by [31], who removed MB with *Nostoc commune* biomass. Likewise, Ouaddari et al. [18] used clay minerals, such as kaolinite and montmorillonite.

The optimal pH for adsorption is between 6 and 8 (Figure 5a). At low pH values (pH < pHpzc), the adsorbent surface is predominantly covered by H^+^ ions, which hinders interaction with the cationic ions of the MB due to electrostatic repulsion and occupation of the active sites. On the other hand, at slightly alkaline pH, the proton concentration decreases and provides greater availability of active sites, facilitating the interaction and adsorption of the dye on the surface of the adsorbent [18,20,68].

Other plant-derived materials have reported higher removal percentages (Table 2), although the adsorbent dose considerably exceeds that used in the present study, reaching doses between 200 and 60,000 mg of adsorbent/L [6,11], with activated carbon being the most notable [8,16]; however, this material requires activation with acidic substances that are chemically attached to other compounds, as is the case reported by Kuang et al. [93], who used doses of 50 and 75 mg/L of a synergistic adsorbent modified activated carbon with cationic surfactant, removing up to about 90%.

### 3.6. Optimization Process

Empirical models involving pH and adsorbent dose were evaluated, with removal percentage as the response. The model coefficients are shown in Table 3. It is observed that the quadratic model with interaction reports a better fit coefficient (*R*^2^ = 0.88), a higher F_cal_ value (12.38), and that most of its coefficients report a *p*-value < 0.05; that is, the model presents a validity of fit for the parameters dose and pH, being able to report a good prediction of the removal percentage [95,96].

The optimal values for the MB removal process were found using the quadratic model (Table 4). These optimal pH and dosage values allowed for the evaluation of MB adsorption kinetics.

### 3.7. MB Adsorption Kinetics

Adsorption kinetics explains the interaction of adsorbate molecules with the surface of an adsorbent over time and involves diffusion, mass transfer, and progressive surface saturation phenomena [97].

The PFO model assumes that the rate at which adsorption sites are occupied is related to the number of available sites [98,99]. This model showed the best fit with an *R*^2^ of 0.99, low values of *ARE* (1.37) and *X*^2^ (0.05), along with random residuals (Table 5), confirming a good ability to describe the process [100,101]. The adsorption capacity was found to be 19.62 mg/g, and the rate constant *k*_1_ was 0.13 min^−1^ (Table 5).

**Figure 6:**
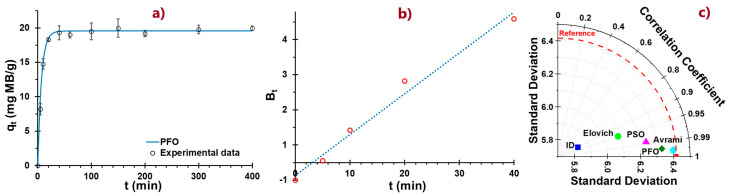
(**a**) MB adsorption kinetics curve, (**b**) Boyd plot: Bt vs. t, (**c**) Taylor diagram for kinetics models.

On the other hand, the PSO model assumes that the adsorption rate depends on the square of the number of free sites and is widely applied to describe MB adsorption on various adsorbents because of its good correlation with experimental data [45]. This model also reported a good fit (Figure 6a), with an *R*^2^ of 0.97, but with relatively high values of *ARE* (5.82) and *X*^2^ (0.85), and slightly biased residuals. Unlike the PFO model, *q_e_* was higher (20.60 mg/g), with *k*_2_ 0.01 (g/mmol-min) (Table 5), this value indicating slower kinetics in general.

The Elovich model also presented an acceptable fit (*R*^2^ = 0.90) with *ARE* values of 6.03, *X*^2^ of 1.27, and random residuals (Table 5). The parameters *α* (200.82) and *β* (0.49) indicate that the initial adsorption process is rapid with moderate surface coverage [102,103].

The ID model indicates that the process rate is limited by the diffusion of adsorbate molecules within the adsorbent pores [48,49]. This model reported an unfavorable fit with lower *R*^2^ (0.42) and high values of *ARE* (15.97) and *X*^2^ (4.61) and biased residuals (Table 5). Therefore, it could be assumed that the NC/ANS adsorbent has low porosity and a heterogeneous surface area.

Regarding the Avrami model (R^2^ = 0.99), k_Av_ = 0.07 min^−1^ was found, suggesting a rapid adsorption process during the first few minutes. This means that NC/ANS has a high availability of active sites that promote adsorbate–adsorbent affinity, confirming the surface heterogeneity of the adsorbent [51]. This same fact is manifested by the Boyd model, where the curve B_t_ vs. t does not cross the origin (Figure 6b), indicating that the external mass transport governs the sorption process [52]. The parameter *n* of the Avrami model, which indicates the type of kinetic mechanism, was reported as 1.26. This value suggests mainly surface adsorption with some internal diffusion, primarily due to NC because of its porous structure. It also indicates the nucleation of MB with the ANS side of the copolymer, which would facilitate adsorption involving both physical and chemical interactions [104]. This duality is advantageous because it allows the use of NC/ANS for removing both anionic and cationic dye residues.

The Taylor plot (Figure 6c) confirms that the Avrami and PFO models fit the experimental data best. In contrast, the Elovich and ID models present a less accurate fit, suggesting that neither surface heterogeneity nor intraparticle diffusion is the predominant mechanism in the adsorption process for this system [105].

Figure 6a represents the adsorption kinetics curve of the best-fitting model (PFO), characterized by a high-speed initial phase where the adsorption capacity increases rapidly, reaching equilibrium within approximately 40 min. Afterward, the adsorption capacity stabilizes at approximately 20 mg MB/g. The good fit of PFO and PSO suggests that adsorption is controlled by physical mechanisms, where the adsorption rate is proportional to the number of available sites, typical of systems where physisorption mechanisms predominate, since physisorption and chemisorption processes occur during adsorption [102,106]. This makes it a material that can be easily regenerated during several cycles, as it maintains its structure by establishing bonds of lower energy, such as Van der Waals forces, with BM.

Thus, the adsorbent shows excellent potential to remove the MB dye, allowing for quick removal of MB through physisorption mechanisms, establishing low-energy chemical bonds, predominantly hydrogen and dipole–dipole bonds, which causes the reversibility and the formation of MB multilayers, making the NC/ANS adsorbent a material with regeneration, which promotes its further study.

These results confirm the potential of the NC/ANS adsorbent as an efficient and stable material for removing cationic dye pollutants from aqueous solutions. The increased availability of its functional groups from the activated clay and activated *Nostoc sphaericum makes* the pH_PZC_ around neutral. This versatility enables it to function in both acidic and basic media, making it suitable for removing anionic dyes. However, the removal of these is lower because the adsorbents studied have greater availability of functional groups with negative charge [107,108]. Furthermore, its nanometric size makes it ideal for use in smaller quantities than other materials of the same origin, whose application rates far exceed those of NC/ANS. Adsorption occurs rapidly within the first 20 min.

### 3.8. MB Adsorption Equilibrium

Studying adsorption isotherms reveals how adsorption occurs at different adsorbent concentrations and how the adsorbent interacts with the adsorbate at equilibrium (maximum adsorption). The parameters of isotherm models enable the design of residual dye treatment systems on a semi-industrial and industrial scale [109,110].

**Figure 7:**
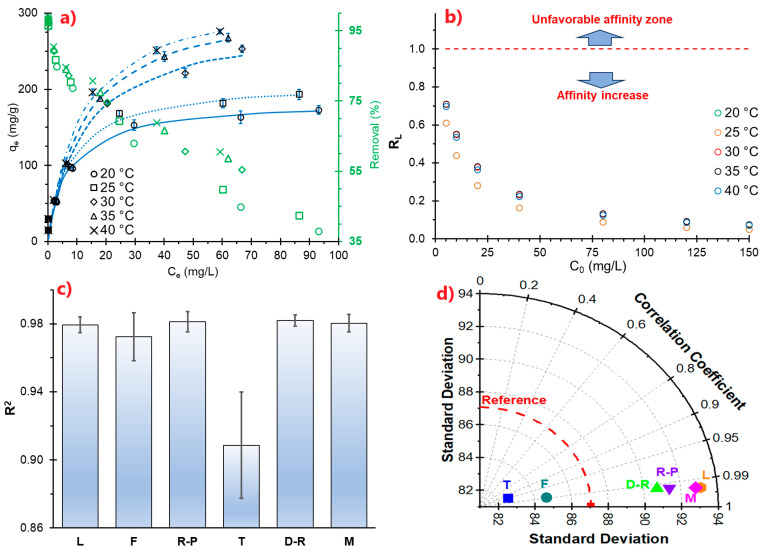
(**a**) MB adsorption isotherm curves by Langmuir model, (**b**) R_L_ separation factor curves, (**c**) fit coefficient of adsorption models, (**d**) Taylor diagram for isotherm models.

The L, M, and DR models demonstrated temperature-dependent saturation adsorption capacities (*q_m_*) at the monolayer level, which increased considerably from 180 to 350 mg/g on average at temperatures ranging from 20 to 40 °C, respectively; this same fact can be evidenced by the relative adsorption capacity (*k_f_*) of the Freundlich molding (F) (Table 6). Similarly, the percentage removal is higher than 90% for low concentrations of MB (10 mg/L), while at high concentrations (150 mg/L of MB), it reports removal of around 60% at 40 °C, compared with 37% at 20 °C (Figure 7a).

This suggests that the MB removal process in an aqueous medium would be better in the range of 30 to 40 °C by using NC/ANS, with favorable adsorption (1.0 < *n* < 1.0 [111]) and high affinity at higher temperatures, evaluated through the separation factor *R_L_* (calculated from *k_L_*) and *A_T_* (Figure 7b and Table 6, respectively), which is characteristic for this type of material [112,113,114].

On the other hand, *k*_2_ of the multilayer model (M) tends to 0.0, which indicates that the MB adsorption process would occur mainly at the monolayer level, with high saturation (1/n < 0) due to the availability of active adsorption sites. This suggests a heterogeneous NC/ANS surface [115], as confirmed by the parameters *a_R_* and *g,* whose values are different or far from the unit [115,116].

The *b_T_* parameter (Table 6) reveals the binding energy of MB in NS/ANS active sites. The obtained values suggest that MB adsorption requires less energy at higher temperatures. This adsorption process is endothermic (ΔH 21.01 kJ/mol, Table 7), which occurs spontaneously and is thermodynamically favorable (ΔS 16.89 J/mol·K), manifesting strong physisorption [112], as indicated by the values of ΔG (between −26.63 and −25.65 kJ/mol) and the adsorption free energy E (between 6.76 and 7.59 kJ/mol, Table 7) [59,60]. These types of processes are required in the implementation of ionic dye treatment systems in wastewater due to the low energy demand required, so NC/ANS becomes a potential option as a wastewater dye adsorbent.

On the other hand, the studied models were adequately adjusted to the experimental data, reporting values of R^2^ > 0.98, with the exception of the Langmuir and Temkin models, as observed in Figure 7d.

### 3.9. MB Adsorption by NS/ANS Regeneration

**Figure 8:**
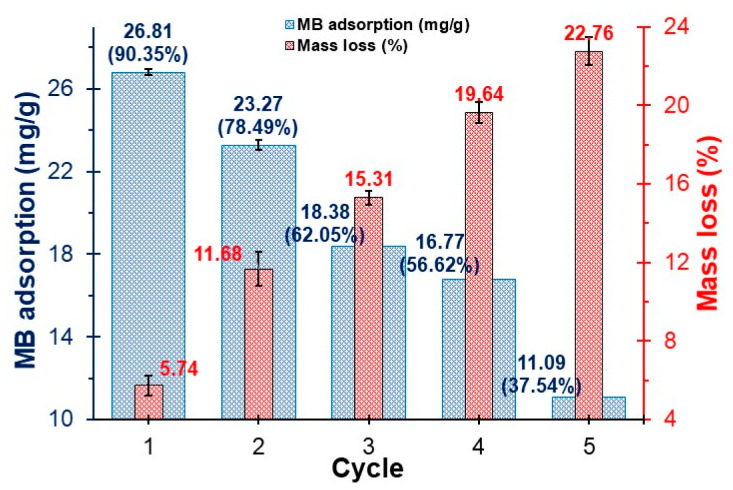
MB adsorption and NS/ANS mass loss after regeneration cycles.

When regenerating an adsorbent, it is important to consider factors such as its structural properties, the conditions of adsorption and desorption, and the chemical interactions between the adsorbent and the dye. Adsorption was observed to be 26.81 mg/g (90.35%) in the first cycle but decreased considerably to 37.54% after the fifth cycle (Figure 8). This phenomenon occurred because the MB adsorbate strongly bound to the active sites of the adsorbent, primarily through chemical adsorption [104], although this is manifested to a lesser extent, as evidenced in the kinetic study. Likewise, the loss of mass during the NS/ANS regeneration process is affected, reaching up to 22.76%.

Another key aspect is the desorbent medium used. Mild acid solutions, such as hydrochloric acid at very low concentrations, minimize denaturation of the adsorbent structure while slightly modifying the pHpzc. This behavior was evident in adsorbent materials derived from clays and biological sources [62,104,117,118].

## 4. Conclusions

Residues of cationic dyes are persistent and toxic to water. The use of environmentally friendly adsorbents is a growing alternative. The study proposed the use of an atomized freshwater alga, *Nostoc sphaericum* (ANS), and activated clay (AN). Characterization of NC/ANS reveals structural and surface properties favorable for MB adsorption. The pH_PZC_ values (pH 3 and 6) indicate a surface with mixed characteristics, attributed to the combination of NC and ANS. ζ indicates good stability with values between −16.32 and −39.34 mV over a wide pH range (3 to 12), with higher affinity to neutral conditions. The particle size ranges from 532.17 to 5468 nm, exhibiting a heterogeneous morphology with lamellar and filamentous structures and featuring the availability of active functional groups (hydroxyl, carboxyl, and amide) that increase the number of active sites available for interaction with methylene blue. Maximum removal of 97.98% was achieved from an initial concentration of 10 ppm MB, pH 6, and 50 mg/L NC/ANS adsorbent. The optimization process resulted in optimum pH conditions (6.8) and a dose of 32.9 mg/L, from which the adsorption kinetics study was carried out, which showed rapid adsorption during the first 40 min of adsorption. The PFO and PSO models fit the kinetic process adequately, reporting adsorption capacities around 20 mg/g, suggesting that physisorption processes control adsorption at the specific active sites. Isothermal models showed that adsorption mainly occurs at the monolayer level and on a heterogeneous surface, with favorable adsorption of high MB saturation in the range of 20 to 40 °C, through an endothermic (ΔH 21.01 kJ/mol), spontaneous, and thermodynamically favorable process (ΔS 16.89 J/mol·K), reporting removal efficiency up to 37.54% after five regeneration cycles. The NC/ANS adsorbent is projected to be an efficient material due to its low application rate, taking advantage of the synergy of activated clay and the hydrocolloid from the algae Nostoc sphaericum, which confers stability during the removal of cationic dyes in aqueous solutions. However, challenges remain, such as its pilot-scale application, the effect of temperature, and competition from other dissolved ions.

## Figures and Tables

**Table 1:** Adsorbent particle size.

pH	Peak 1 (nm)			Peak 2 (nm)		
	$\bar{x}$	(±) S	CV	$\bar{x}$	(±) S	CV
2	1057.33	38.85	3.67	-	-	-
3	889.93	88.06	9.89	-	-	-
4	651.83	19.67	3.02	5468	8	0.146
5	677.47	82.81	12.22	-	-	-
6	674.37	25.27	3.75	-	-	-
7	627.9	77.36	12.32	5333	49.5	0.93
8	645.7	68.93	10.68	-	-	-
9	663.63	48.84	7.36	5334	9	0.169
10	686.37	39.69	5.78	-	-	-
12	532.17	95.52	17.95	2716.8	3632	133.69

where $\bar{x}$ is the mean, S is the standard deviation, and CV is the coefficient of variation (%).

**Table 2:** Removal percentage (R) and adsorption capacity (*q_e_*).

Treatment	Initial MB Concentration (mg/L)	pH	NC/ANS Dose (mg/L)	Adsorption (mg/g)				Removal (%)				Source
				$\bar{x}$	± S	CV	*	$\bar{x}$	± S	CV	*	
T1	10	5	20	2.73	0.15	5.38	a	72.59	1.48	2.03	d	
T2	10		30	1.34	0.19	14.5	b	86.53	1.95	2.26	c	
T3	10		50	0.26	0.02	5.93	c,d	97.38	0.16	0.16	a,b	
T4	10	6	20	1.25	0.05	3.83	b	87.48	0.48	0.55	c	
T5	10		30	0.38	0.01	3.2	c	96.19	0.12	0.13	b	
T6	10		50	0.2	0.05	24.7	d	97.98	0.5	0.51	a	
T7	10	8	20	1.19	0.02	2.04	b	88.01	0.24	0.28	c	
T8	10		30	0.34	0	0.99	c,d	96.57	0.03	0.04	a,b	
T9	10		50	0.34	0.04	12.9	c,d	96.59	0.44	0.46	a,b	
Activated carbon	25	7	2000					95				[6]
Clay	25	7	2000					89				[6]
Fly ash	25	7	2000					95				[6]
Pine cone	25	7	2000					99				[6]
Algae cells	10	6	3000	21.6				93				[7]
Activated carbon	10	6	5000					99.1				[8]
Activated carbon	50	7	200					99				[9]
Activated carbon	50	7	1000					~100				[10]
Green Algae	25	7	60,000					94.3				[11]
Green macroalgae	10	5	1000	25.5								[94]
Activated carbon modified by anionic surfactants	10	8	50					~80.0				[93]
	10	8	75					~90.0				[93]

where $\bar{x}$ is the arithmetic mean, S is the standard deviation, and C.V. is the coefficient of variability (%). * Letters indicate significant differences between treatments, assessed using the Tukey test at the 5% significance level.

**Table 3:** Coefficients of empirical models for MB removal (%).

Model	$\beta_{0}$	a	b	c	d	e	$R^{2}$	F_cal_
Linear	61.23	0.45	2.35	-	-	-	0.52	5.32
*p*-value	0.00	0.03	0.18					
Linear with interaction	27.55	1.46	7.67	−0.16	-	-	0.58	4.70
*p*-value	0.35	0.11	0.12	0.23				
Quadratic with interaction	−120.51	3.44	44.69	−0.16	−0.03	−2.82	0.88	12.38
*p*-value	0.04	0.03	0.05	0.09	0.04	0.08		

where $\beta_{0}$, intercept; a, dose coefficient; b, pH coefficient; c, pH and dose coefficient; d, dose coefficient squared; *e*, pH coefficient squared.

**Table 4:** Optimal parameter values for MB removal.

Parameters	Min	Max	Optimum
Dose (mg/L)	20	50	32.9
pH	5	8	6.8
Removal (%)			99.1

**Table 5:** MB adsorption kinetic model parameters.

Model	Parameters		Statistical Indicators	
Pseudo-first order			*R* ^2^	0.99
	*q_e_*	19.62	ARE	1.37
	*k*_1_	0.13	X^2^	0.05
			Residuals	Random
Pseudo-second order			*R* ^2^	0.97
	*q_e_*	20.6	ARE	5.82
	*k*_2_	0.01	X^2^	0.85
			Residuals	Slightly tendentious
Elovich model			*R* ^2^	0.9
	*α*	200.82	ARE	6.03
	*β*	0.49	X^2^	1.27
			Residuals	Random
Intraparticle diffusion			*R* ^2^	0.42
	*C*	13.97	ARE	15.44
	*k_i_*	0.39	X^2^	4.61
			Residuals	Tendentious
Avrami	*q_e_*	*19.50*	*R* ^2^	0.99
	*k_Av_*	*0.07*	ARE	1.79
	*n*	*1.26*	X^2^	0.07
			Residuals	Tendentious

where $q_{e}$ is the amount of MB adsorbed (mg/g); $k_{1}$ is the adsorption rate constant of the PFO model; $k_{2}$ is the adsorption rate constant of the PSO model; *R*^2^ is the fit coefficient; *ARE* is the mean relative error; *X*^2^ Chi-Square.

**Table 6:** The MB adsorption isotherm model parameters onto NC/ANS.

°C	L		M			F			RP			T		DR	
	*q_m_* (mg/g)	*k_L_* (L/mg)	*q_m_* (mg/g)	*k*_1_ (L/mg)	*k*_2_ (L/mg)	*k_f_* (mg^1−1/n^/L^n^)	1/*n*	*n*	*k_R_* (L/g)	*a_R_* (L/mg)	*g*	*b_T_* (J/mol)	*A_T_* (L/mg)	*q_m_* (mg/g)	*K_DR_* (mmol^2^/J^2^)
20	184.942	0.137	184.647	0.137	≈0.0	45.256	0.310	3.229	28.796	0.190	0.956	94.084	7.199	195.147	0.009
25	210.307	0.128	210.307	0.128	≈0.0	49.196	0.322	3.110	25.374	0.108	1.024	86.678	7.792	222.767	0.009
30	289.089	0.082	220.440	0.132	≈0.0	48.005	0.400	2.499	42.036	10.453	0.617	70.780	7.419	305.489	0.011
35	318.748	0.081	283.049	0.098	≈0.0	51.184	0.412	2.426	35.174	0.208	0.854	67.696	8.169	340.427	0.011
40	330.153	0.087	330.148	0.087	≈0.0	54.960	0.408	2.448	28.669	0.087	0.999	66.932	8.899	356.349	0.010

where L is the Langmuir model; M is the multilayer model; F is the Freundlich model; RP is the Redlich–Peterson model; T is the Temkin model; and DR is the Dubinin–Radushkevich model.

**Table 7:** Thermodynamic parameters of MB adsorption process onto NC/ANS.

Energy Type		20 °C	25 °C	30 °C	35 °C	40 °C
Gibbs free	ΔG (kJ/mol)	−26.04	−26.32	−25.65	−26.04	−26.63
Adsorption free	E (kJ/mol)	7.59	7.53	6.76	6.77	6.95
Entropy	ΔS (J/mol·K)	16.89				
Enthalpy	ΔH (kJ/mol)	21.02				

## Data Availability

The original contributions presented in this study are included in the article. Further inquiries can be directed to the corresponding authors.
